# Establishing and Sustaining an ECPR Program

**DOI:** 10.3389/fped.2018.00152

**Published:** 2018-06-06

**Authors:** Peter C. Laussen, Anne-Marie Guerguerian

**Affiliations:** ^1^Department of Critical Care Medicine, Department of Anaesthesia, The Hospital for Sick Children, University of Toronto, Toronto, ON, Canada; ^2^Department of Critical Care Medicine, Department of Paediatrics, University of Toronto, ON, Canada

**Keywords:** extracorporeal life support, cardiac arrest, cardiopulmonary arrest, resuscitation, reliability, resilience

## Abstract

The use of extracorporeal support after failed return of a spontaneous ciruculation during cardiopulmonary resuscitation (ECPR) is well described. There are 4 distinct phases for resuscitation with ECPR and the time spent in each phase is critical for successful outcome. Recommendations for ECPR previously published by the American Heart Association provide the context for implementing a consistent and well-rehearsed system for ECPR, by people with the knowledge, experience and resources to deploy ECPR in the most optimal time frame possible in selected patient populations. In this manuscript we review the current status of ECPR for acute cardiac failure and the components we believe are necessary to develop and sustain a reliable and resilient program.

## Patient selection

The use of extracorporeal support after failed return of spontaneous circulation during cardiopulmonary resuscitation (ECPR) is well described in children and in adults (1–18). The utility of ECPR has been demonstrated in single center and registry retrospective studies. The most recently published registry data from 2011 to 2015 from the Extracorporeal Life Support Organization (ELSO), reported a survival to discharge using ECPR of 40% in neonatal and pediatric populations (4). The survival after ECPR is lower in the adult population reported at 28%, and while this may reflect different characteristics of the patients selected for ECPR such as predominantly having ischemic heart disease, there may be systems issues that impact outcomes and the timely use of ECPR (e.g., out-of-hospital location).

ECPR for refractory cardiopulmonary arrest can be applied for the purpose of supporting a patient for cerebral-cardiopulmonary resuscitation (cerebral-CPR) (19) or for the purpose of supporting organs for donation (20). This paper focuses on using ECPR for cerebral-CPR. In this context, ECMO technology is generally applied for the purpose of (1) *bridge-to-recovery* by providing time for diagnostic procedures and/or therapeutics to be delivered (e.g., acute arrhythmia following cardiac surgery, or from electrolyte disturbance with loss of cardiac output and refractory to conventional therapy, acute myocarditis with complex arrhythmias or heart block, patient with residual lesions after cardiac surgery who could undergo additional surgery or intervention in the catheterization laboratory); (2) *bridge-to-organ transplantation* or as a means to bridging to decision with another device—to consider organ transplantation; or for (3) *bridge-to-decision*, which includes decision to continue, decision to stop advanced technological support, and to bridge to palliative care plan (21).

The most important decision regarding ECPR relates to patient selection. Programs offering ECPR must have predefined selection criteria for patient groups (e.g., in-hospital cardiac arrests or out-of-hospital cardiac arrests) where extracorporeal technologies are expected to provide an added value to the quality of CPR. Protocols are then developed and operationalized for these groups. Within these patient groups, individual patient selection decisions may be difficult to make at the actual time of the resucitation and prolonged discussions will waste time. Therefore it is necessary that discussions be held pre-emptively in high risk patients, in order to allow balanced decisions to be made to use or not use ECMO in the context of a cardiac arrest (which is different than using ECMO for cardiopulmonary failure), and to predefine the type of surgical cannulation that will optimize neurologic and cardiac reperfusion. It is also reasonable not to apply ECMO during resuscitation when it is known—or at least likely—that there will be no direct benefit to the individual patient.

Anticipating the risk for a cardiac arrest and appreciating an evolving low cardiac output state with impaired oxygen delivery requiring escalation of care, should trigger the discussion about *pre-arrest* ECMO support, rather than waiting for a cardiac arrest to occur and deploy ECMO during acute CPR. The indications and thresholds for pre-arrest ECMO vary between insititutions and within patient populations. More studies are required in this area, but we forsee that within the emerging era of Artificial Intelligence and “Big Data,” it will be possible to model individual patient risk and provide clinicans with additional information for making decisions (22–25).

### Pediatrics

In our institution and in other similar organizations with established extracorporeal life support (ECLS) programs, there is sufficient data to support the benefit of ECPR in children with cardiac disease who have an in-hospital cardiac arrest (see below) (3, 11, 13, 16, 26–31).

The most recent American Heart Association CPR and Emergency Cardiovascular Care Guidelines that integrate 2010 (in- and out-of-hospital cardiac arrests) and 2015 (in-hospital cardiac arrests) recommend the following: “*ECPR may be considered for pediatric patients with cardiac diagnoses who have in-hospital cardiac arrest in settings with existing ECMO protocols, expertise, and equipment (Class IIb, LOE C-LD)*.” There is however, insufficient published data to support the benefit of ECPR over conventional CPR in all pediatric cardiac arrest events (32–35).

In our organization, an individual selection process is applied to children *without primary heart disease* who have an in-hospital cardiac arrest. This is based on whether the bridging application of extracorporeal technology will assist in supporting a reversible condition and the quality of resuscitation measures (e.g., refractory hyperkalemic cardiac arrest in a child with tumor lysis syndrome), or where the bridging application is part of a predefined care plan (e.g., cardiac arrest in a child with end-stage pulmonary arterial hypertension listed for lung transplantation suitable for bridge to transplantion with extracorporeal life support and conventional CPR is not expected to be effective) (36).

Pediatric patients with significant disease co-morbidity such as end organ dysfunction and those with chromosomal abnormalities have the highest risk for mortality during extracorprcorporeal support, and it is reasonable for these patients to not receive ECPR in the context of a cardiac arrest event (5, 8). Kane et al reported on the outcomes following ECPR in a large, single-center, retrospective study, and noted that known non-cardiac and chromosomal anomalies were important factors contributing to adverse outcome (OR 3.2, 95% CI 1.3–7.9). Discussion about the suitability for ECPR should be possible in the majority of pediatric patients as ECPR is predominantly an in-hospital and ICU event. The ELSO 2016 International Report of 1828 pediatric ECPR cases over the period 2011–2015 noted that most of the cardiac arrests supported with ECMO occurred in the Intensive Care Unit (72%), and only 3% in the emergency department and 5% on the ward setting (4).

### Pediatrics: cardiac

Newborns, infants and children after congenital cardiac surgery have a 10-fold higher risk for an in-hospital cardiac arrest after cardiac surgery, and their survival to discharge following the use of ECPR has been reported at higher than 50% (13, 31, 37). The Hospital for Sick Children ECLS Program has offered ECPR since 2000 (38), and since 2009, 148 patients have received ECPR in the Cardiac Critical Care Unit with 54% survival to discharge. Based on local experience, registry data and retrospective studies, patient selection is a key factor responsible for improving survival over the years. Successful outcome is influenced by the type of cardiac disease. Single ventricle patients being at higher risk for cardiac arrest and with worse outcomes with the use of ECPR (39). Patients with acute fulminant myocarditis are some of the most suitable candidates for ECPR, as this is often a favorable example of a reversible acquired cardiac condition (40, 41).

Performing high quality CPR is essential no matter the indication for ECPR. It is important to appreciate that in some conditions, specifically in patients with congenital heart disease, the effectiveness of conventional CPR is limited. New recommendations for the resuscitation of children with heart disease have been published (42), and there are three functional considerations that limit the effectiveness of conventional CPR in children with congenital heart disease.

- *Limited stroke volume* with chest compressions, such as from:
  - Atrio-ventricular or semilunar valve regurgitation, or
  - Restrictive myocardium
- *Limited effective pulmonary blood flow and oxygenation with compressions*, such as from:
  - Pulmonary outflow obstruction,
  - Elevated pulmonary artery pressure or pulmonary vascular resistance, or
  - Cavo-pulmonary connection
- *Limited cerebral perfusion*, such as from:
  - Cavo-pulmonary connection with elevated superior vena cava pressure, or
  - Semilunar valve regurgitation or aorto-pulmonary run off across a shunt or collateral vessels

While these circumstances can limit the effectiveness of conventional CPR they may not alone be contraindications for ECPR. There are no specific recommendations in these circumstances to modify the technique for conventional CPR from that recommended for patients with structurally normal hearts. However, it is important these functional considerations are discussed and appreciated before and during resuscitation, and these place even more pressure on ensuring the system for ECPR is optimal for the performance of each of the phases of ECPR described below. There is variability in practice between institutions that use ECPR for pediatric patients with heart disease; there is a huge opportunity to gain knew insights on what protocols and interventions ensure the optimal quality of extracorporeal cerebral-CPR (11).

Given the functional CPR considerations and the importance of the system's in place, the 2015 Pediatric Advanced Life Support Guidelines (34) and the American Heart Association Scientific Statement include recommendations for children with heart disease for the setting where ECPR is undertaken (42); specifically: “*If cardiac arrest develops in the child with heart disease and there is no prompt return of circulation, it is reasonable to initiate ECPR (Class IIa; Level of Evidence C), and that ECPR can be most effectively deployed in locations with rapid access to ECLS equipment, skilled ECLS personnel, and adequate space to accommodate a large team (Class IIa, LOE C).”*

### Adults

The most recent 2015 American Heart Association CPR and Emergency Cardiovascular Care Guidelines for *Adult Advanced Life Support* (43) target ECPR applications where the technology may allow additional time to treat adults with reversible causes of cardiac arrest (e.g., acute coronary artery occlusion, pulmonary embolism, refractory ventricular fibrillation, cardiac injury, myocarditis, cardiomyopathy, congestive heart failure, drug intoxications), or serve as a bridge to another extra-or paracorporeal ventricular assist device. These recommendations are the following: “*There is insufficient evidence to recommend the routine use of ECPR for patients with cardiac arrest. In settings where it can be rapidly implemented, ECPR may be considered for select cardiac arrest patients for whom the suspected etiology of the cardiac arrest is potentially reversible during a limited period of mechanical cardiorespiratory support. (Class IIb, LOE C-LD).”*

In adults, the contemporary literature reports increasingly the deployment of ECPR in both out-of-hospital and in-hospital cardiac arrest. The variability between studies and settings limits estimating the probability of favorable outcome (which ranges from less than 10% to greater than 40%). The populations studied have an arrest that is suspected to be of cardiac origin and where the event is witnessed (26, 44–46). Adult programs reporting improved outcomes have refined patient selection criteria and high performing response systems that may also include coordination with interventional cardiology systems (47–50).

### Out-of-hospital ECPR

The key features of out-of-hospital systems include clear inclusion criteria with rapid protocol-driven emergency systems that either retrieve to cannulate in-hospital or more recently cannulate on-site (49, 51–53). An emergency response team for out-of-hospital cardiac arrest, which includes a doctor, nurse and paramedic, have reported 156 out-of-hospital patients who have been placed on ECMO between 2011 and 2015; as they've evolved their selection protocol and training, the survival has increased from 3 to 38% which is equivalent to the ELSO Registry for pediatric and newborn patients (54, 55). This is a unique and highly trained and resourced team with outstanding communication systems across a large metropolitan area. Such a centralized system with highly trained personnel is beyond the capability of most cities across the world at this time but may well be a model for the future. Some are studying such protocols in randomized trials that bundle several resuscitation measures (mechanical CPR devices, hypothermia therapy) with extracorporeal systems (56). While these trial results may not be generalizable to different regional systems, they will be important for the field.

## ECPR: systems and resources

**Figure 1:**
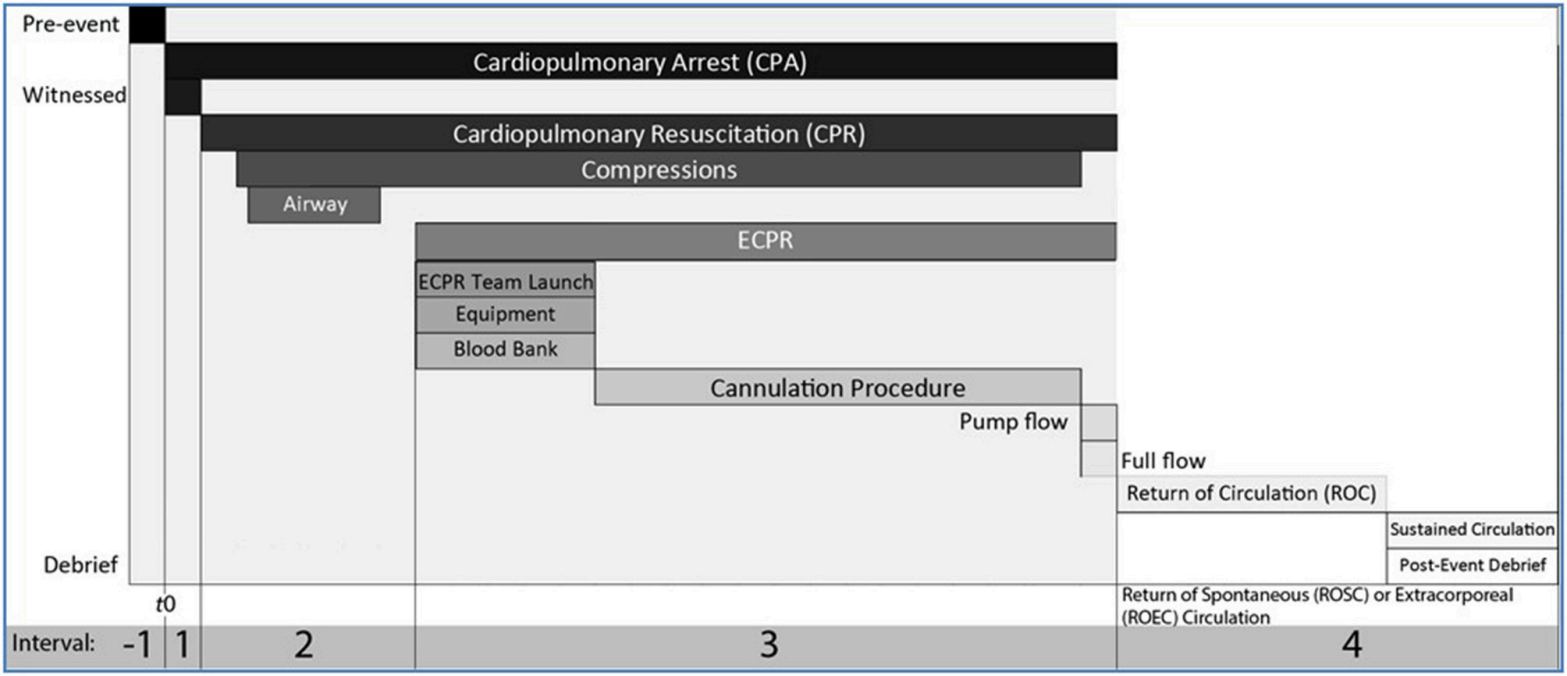
Intervals of resuscitation phases with conventional CPR and ECPR. A cardiac arrest event can be deconstructed in a pre-event interval (Interval −1), and 4 intervals following *T* = 0, the start of cardiopulmonary arrest (CPA). Interval 1 is the interval between the start of cardiopulmonary arrest and start of conventional cardiopulmonary resuscitation (CPR) measures, ideally less than 1 min. Interval 2 starts with the start of conventional CPR which includes C-A-B or A-B-C and if needed early defibrillation. During Interval 2, with ongoing high quality CPR, if there is no return of spontaneous circulation (ROSC) or the likelyhood of ROSC is low or if there are functional considerations that limit the effectiveness of conventional CPR, the decision to call for ECPR must be made (5–10 min from *T* = 0). Interval 3 starts with the launch of ECPR, while ongoing high quality conventional CPR continues. During Interval 3, a group page is used to deploy the team, prepare the cannulation location, notify the blood bank, move the patient in the correct location, position, prepare anatomical site for cannulation, cannulate artery and vein, prepare clear primed circuit. If there is no ROSC with ongoing conventional CPR, ECMO flow is started, pump flows are increased gradually. Interval 3 stops and Interval 4 starts when target flows are achieved to return of extracorporeal circulation (ROEC). If ROSC occurs during Interval 3, conventional CPR stops, and the intensive care physicians decides if the pharmacological support is sufficient to continue during the post-cardiac arrest phase, or if VA ECMO cannulation should still continue. A post-event debrief session is conducted after ROC (either once sustained ROSC or ROEC). Ideally Intervals 1+2+3 are 30 min or less.

There are 4 distinct *intervals* that delineate phases of care when applying ECPR, Figure 1. The steps and the duration of each phase is critical for a successful outcome. The pathophysiology of the clinical state leading to the arrest (before time *T* = 0) may be relevant to individual outcomes but we focus here on the intervals of care relevant to evaluating the system. These include the following:

Interval 1: Interval from start time of cardiac arrest (*T* = 0) to start of conventional CPR
Interval 2: Interval from start time of CPR to launching the ECPR system
Interval 3: Interval from time of launch of ECPR to achieving return of circulation with adequate flow and perfusion
Interval 4: Interval from time of return of circulation to on-going targeted post-cardiac arrest care

Keys components to minimize ischemia and reperfusion injury include:

1. **Limiting the Time of No-flow Cardiac Arrest** (see Interval 1, Figure 1): Witnessed and in-hospital cardiac arrests offer the best conditions where the period of no-flow prior to CPR starting is kept to a minimum to increase the chance of myocardial recovery and reducing the risk for ischemic brain injury. It is more difficult to assess in the out-of-hospital setting, where the effectiveness of bystander CPR is difficult to guage. Currently, in our practice at The Hospital for Sick Children, we do not deploy ECPR if the cardiac arrest was un-witnessed and occurs out-of-hospital. Ideally, Interval 1 is less than 1 min.
2. **Excellent Conventional CPR**: (see Intervals 2 and 3, Figure 1) this means that the time between cardiac arrest to first resuscitation measures (ABC or CAB) with chest compressions is minimized at < 1 min and that the recommendations for one- or two-person CPR, airway management, and effectiveness of compressions are closely followed. In the in-hospital setting this will include the addition of continuous monitoring with ECG and plethysmography, continuous end-tidal CO_2_ monitoring as an indicator of pulmonary blood flow, and intra-arterial blood pressure monitoring when available. Cerebral and somatic near infrared oximetry (NIRS) may have a role in assessing oxygen delivery during compressions but this yet to be validated. Bilateral cerebral and/or peripheral NIRS are standard monitoring in patients on ECMO in our organization to monitor reperfusion and are applied as soon as feasible.
3. **Location, Launching ECPR and Cannulation**: ECPR should occur in a well controlled environment in which protocols are in place. This is critical. Time is of the essence for ECPR, from the moment of deployment to the start of ECMO flows and return of extracorporeal circulation (ROEC). There can be a sense of chaos if ECPR is undertaken in an unfamiliar environment, looking for equipment, for access to medical gases, surgical or technical support, and finding adequate space. All of this must be worked out well in advance and adhred to during deployment of ECPR.

- **Protocols**: Proper institutional or regional protocols include decision pathways or flow diagrams that indicate the “who, what where” to launch ECPR, and indicate where the cannulation will be undertaken and circuit started. Each system should test alternative locations (e.g., with or without simulation). It many circumstances, it may be preferable to *transport the patient with ongoing CPR to the cannulation location* rather than transport the resources (people and equipment) to the patient.
  In most pediatric institutions, the equipment needed and personel experienced with ECLS are in the intensive care units. It takes unnecessary time to mobilize these resources and cannulate in an unfamiliar environment, such as the emergency department or inpatient ward or out-patient clinic setting. It takes no less time to continue effective and monitored CPR on route to the intensive care unit, and indeed may allow for faster cannulation times as the equipment and personnel can be mobilized and ready for immediate cannulation on arrival to the intensive care unit. Institutions will need to evaluate their performance based on their local setting. At The Hospital for Sick Children, in-hospital cardiac arrests that occur in the operating rooms or image guided therapy location stay in those areas for cannulation; for any other in-hospital cardiac arrest, the patient is transported to the intensive care unit for cannulation.
  For adult ECPR applications, where the options for cannulation are wider and are often performed percutaneously, the location for cannulation for an in- or out-of hospital arrest can be in the emergency departments, in the interventional catheterization suites, in the operating rooms, and in the intensive care units. It is important systems compare processes and outcomes between these options.
  Protocols need to be re-evaluated whenever there is a change to a facility, such as new construction. Similarly, new hospital designs must include a careful and thoughtful understanding of vital patient safety nets, such as how to make sure personnel and resources are able to reach patients during urgent events like cardiac arrest, and facilitate expeditious transfer of patients to resources such as ECPR. These are clinical decisions and they must be made by clinical teams rather than being asked or expected to adapt to constraints placed by new building design.
- **Equipment**: The cannulation equipment for ECMO must be available, in prepared packages (bundled) and consistent; it is a waste of time to send staff off to find specific and often unnecessary instruments. Different approaches may be used to cannulate for veno-arterial ECMO during CPR (e.g., peripheral or open chest; open surgical, percutaneous, combination). These may not provide similar cerebral and myocardial reperfusion or may not be achieved by the same individuals (surgeons vs. non-surgeons), but most institutions will pre-define a limited set of default cannulation approaches in order to optimize speed, performance and minimize complications. As examples: a patient with recent sternotomy after cardiac surgery will be cannulated relatively faster by reopening the chest rather than via a peripheral cannulation site, but the tradeoff may be interruption to cardiac compressions during cannulation. Medical patients less than 10–15 kg will be more rapidly cannulated via the carotid and jugular right neck approaches while a 50 kg adolescent may be cannulated faster via femoral artery and vein on the side optimal to the operator. Therefore, when deciding where to launch ECPR, surgical requirements for cannulation are essential to consider; and these are procedures that should be performed in conditions as close as possible to that of the operating room.

The set up and initiation of the ECMO circuit should be identical each time, and should only vary according to the size of the patient. The selection of arterial and venous cannulae is based on size and blood flow targets. The current use of centrifugal pumps and membranes with low circuit volumes means that the circuit can be clear-primed and ready to deploy within 10–15 min and therefore should not be a limitation to establishing flows. The circuit can be set up away from the bedside and brought forward once cannulae have been placed. There should not be any delay in establishing flow and perfusion by waiting for blood to become available; a clear crystalloid prime is likely to be used in most circumstances. An exchange transfusion can occur once on ECMO support to elevate the hematocrit to the desired level (35 to 40%) with type-matched blood. Some pediatric centers titrate the FiO_2_ in the ECMO circuit and use temperature targeted therapy of 34°C (hypothermia) or normothermia; however there is no comparative data suggesting one is superior to the other.

## Personnel experienced in ECLS

Important in any setting, proper protocols ensure that multiple roles, responsibilities, and tasks are concurrently being completed, and these should not be improvised. We include in Figure 1 an example of the multiple streams of actions that need to be completed concurrently. This is probably the most important component for any ECPR deployment. Leadership, orchestration and role assignment are key.

- **Decision to call**. The *decision to call* and launch ECPR should be made as early as possible, ideally within the first two rounds of resuscitation medicines having been administered. Some systems will use clock time and in our organization, we expect the physician to make the decision to call within 5-10 min. There needs to be clear lines of authority for calling for ECPR and this should be part of the agreed upon protocol established by the team before implementing an ECPR program. Indecision will lead to unnecessary delays in deployment as well the potential for unnecessary conflict and uncertainty. It is easier to call off deployment if there is spontaneous recovery of the circulation rather than delaying a definitive decision. At The Hospital for Sick Children, the intensive care physician will make the decision to call for ECPR on most occasions. If a cardiac arrest occurs in the operating room or during a cardiac interventional procedure, than the cardiac surgeon or the staff cardiologist or cardiac anesthesiologist will take responsibility for launching ECPR.
- **Whom to call**. It is essential a robust fan-out list is established to make sure the right people are notified for ECPR (End of Interval 2 and start of Interval 3, Figure 1). This fan-out could be either by group page or chat group application. The list should include the surgeons who will be cannulating and ECMO specialists/perfusion specialists, and include information about the cannulation location and size of patient.
- **Availability**. A concern raised for ECPR is the availability of cannulating physicians (e.g., surgeons) and ECMO specialists or perfusion specialists particularly out of hours. This should not be a limiting step, and part of the ECPR implementation is to make sure that in-house staff and teams are trained to start to prepare the patient for cannulation. During this time, it is essential that effective CPR continue and be closely monitored. As to the availability of ECMO specialists or perfusionist to prime and manage the circuit, ideally, there should be in-house trained staff to do this 24/7. It is up to the individual programs as to how this is achieved and who is trained for this role, but whatever approach is taken, it must be consistent with regular training and simulation. This is particularly critical for programs that may have <5–10 ECPR launches per year.
- **Role assignment**. There should be no confusion regarding assigned roles for staff during ECPR. Initial measures already involves assigned roles and responsibilities with a resuscitation leader. In addition, added roles and responsibilities are required for the ECMO deloyment, and parallel concurrent tasks need to be accomplished. An effective system will incorporate:

- An “Inner” manager who coordinates ongoing resuscitation and CPR throughout cannulation.
- An “Outer” manager who can orchestrate all of the support, equipment for the performing cannulation, and very important, control the inevitable crowd, noise and traffic (NB, the ELSO Specialist Manual and Redbook provides several examples) (57). All staff should know their role and understand their set of responsibilities to avoid loss of time, confusions, and wasted time on duplicating tasks.
- Communication must be direct and closed-loop; if anybody sees a discrepancy or problem, they must feel safe and compelled to speak up. This can only come with regular multidisciplinary and structured team training and simulation.

## Duration of resuscitation measures before achieving return of circulation

In the ELSO 2016 International Report, the median duration of ECPR was 40 min (IQR 25, 61 min). Reduced survival for ECPR was associated with lower post-ECMO arterial pH, higher lactate, and end organ injury, and this could reflect the underlying disease and functional state that limits effective CPR, but could also reflect the time it took to cannulate and achieve adequate flows on ECMO. While it is true that survival has been reported after >50 min of ECPR, experienced centers with a well-designed and trained in-house teams are able to reduce the ECPR time to <30 min.

A longer duration of ECPR is associated with adverse neurologic outcome. Lasa et al reported on ECPR during in-hospital cardiac resuscitation from the American Heart Association's (AHA) Get with the Guidelines Resuscitation Registry. For children with in-hospital CPR > 10 min duration, ECPR was associated with improved survival to hospital discharge (OR 2.80, 95% CI 2.13–3.69) and survival with favorable neurologic outcome (OR 2.64, 95% CI 1.91–3.64) when compared to conventional resuscitation. In our organization, we aim to achieve return of circulation (ROC) by 30 min either by spontaneous circulation (ROSC) and conventional CPR or by return of extracorporeal circulation (ROEC) with ECPR; we use this as a performance measure to benchmark the processes applied during resuscitation and ECPR launches. This 30 min is not used to stop resuscitation measures but is used to evaluate lag times for each phase and determine what could have contributed to delays in achieving ROC (e.g., steps completed early during interval 2 such as defibrillation or airway control vs steps completed during interval 3 like vessel cannulation).

Decisions to stop resuscitation or stop attempts at ECPR can be challenging for the physician who does not apply these decisions often. Programs that offer ECPR must be able to stop resuscitation measures and transition to palliative care or continue intensive care with or without advanced technologies. At The Hospital for Sick Children, while cardiac surgeons or the staff cardiologists or cardiac anesthesiologists can launch the deployment of ECPR outside of the intensive care units, the intensive care physicians are paged with the ECPR team launch and are expected to lead or facilitate the decision making process when stopping measures are reasonable. Providing palliative care following the launch of advanced technologies happens often during the post-arrest care, and is part of the comprehensive care due to these patients and their families.

## Post-cardiac arrest care with ECPR management

A notable finding in the recent ELSO report was that therapeutic hypothermia was used after nearly 60% of ECPR events (4). While the THAPCA trial indicates that there is no additional benefit for targeted temperature control with hypothermia after a cardiac arrest over normothermia (58, 59) there is no data yet for the benefit or risk of hypothermia induced *during* the resuscitation or ECPR. Our practice since 2000 has been to initiate ECPR at 34°C and either maintain targeted temperature at 34°C or rewarm at 36–37.5°C based on the individual patient characteristics.

The target flow rate and perfusion pressure should be discussed once ECMO flow has been established and this will vary according to the diagnosis and age of the patient. Cerebral and myocardial reperfusion may be associated with altered cerebral autoregulation and myocardial stunning. Hence, careful attention is required to maintain an adequate cerebral perfusion. The ECMO specialist are trained to carefully increase circuit blood flow to target perfusion pressures, and to carefully titrate gas flow to avoid hypocapnia and hypercapnia, all in an effort to minimize secondary ischemia. Because of the risk for free O_2_ radical formation, we use an ECMO system that allows titration of FdO_2_ (diffusion of oxygen) in the ECMO membrane to be as low as possible to maintain a post membrane arterial PO_2_ above 100–150 mmHg and decrease the FiO_2_ through the mechanical ventilator to room air (or as low) as possible when ECMO flows are initiated. Monitoring with NIRS and transcranial Doppler ultrasonography may be beneficial in this regard.

Once ECMO flow has been established, critical questions about myocardial recovery will need to be addressed. It is common for the myocardium to have electrical activity but with limited ejection after ECPR, and it can take up to 48 h to recover effective ejection and pulse width on the arterial waveform. It is vital that the heart not be over distended as this will cause further myocardial injury from the elevated wall stress. The assessment of need for decompression of the left ventricle, either transcatheter or surgical, can be made clinically at the bedside and with echocardiography.

ECPR involves the exposure to anticoagulation. The risk of intracranial hemorrhage in ECPR is higher than in patients support with ECMO. We speculate that multiple factors may be responsible for this difference (e.g., altered neurovascular unit, anticoagulation practices, cannulation strategies, microemboli, inflammation), but more work is needed to understand what factors are modifiable. Systems using ECPR should have the capacities to provide neuroimaging and neurologic assessments (including electrophysiology, clinical, monitoring) either during and following separation from extracorporeal technologies. A detailed review of the neurologic and functional assessment is beyond the scope on this specific paper, but should be aligned with other post-arrest care guidelines by resuscitation councils for pediatric or adults.

### Reliability and resilience

A *reliable* program is able to consistently respond in a well structured and efficient manner to the need at hand. A *resilient* program is able to adapt to uncertain circumstances when necessary, and recover from unexpected events. Both of these should be charcteristics of a well-functioning ECPR program. To achieve this requires practice, briefing and debriefing, and commitment of individuals and the team as a whole.

### Getting started

Starting a program can be daunting. The first step is to achieve buy-in at a senior administrative level that an ECPR program is a requirement, a fundemental safety net for select patient populations. Given the wealth of data available, there should be no need to prove “value,” nor to present data around a “return on investment.” We acknowledge that the equipment, resourses and training are expensive, but the value is in lives saved and in the fundemental support required to develop a contemporary resuscitation program, particularly in children undergoing cardiac surgery.

The next step is to identify key stakeholders and their contributions to the ECPR program. Leaders should be chosen who have the responsibility and authority to direct all phases of the program. At a minimum this includes the surgeons who will perform the cannulation, critical care clinicians and perfusion or ECPR specialists who will manage the circuit. Settling on equipment and resources to provide ECPR is the next step. It may take 6 months to be prepared, and it is highly recommended that new progams contact and partner with established programs to help navigate all the steps required to establish an ECPR program. These partnerships are critical for sharing information and ideas, and for benchmarking outcomes. In addition, joininig established registries, such as the Extracorporeal Life Support Organization, or the American Heart Association Get with the Guidelines for Reuscitation are recommended as these will also provide important benchmarks against which to measure success.

### Maintaining an ECPR program

The key to maintaining a successful program are consistent cycles of measurement, review and training. Simulation training is particularly important to assess skills, trouble-shoot potential problems and complications that can occur during resuscitation, cannulation and with the circuit, and to ensure the team is well trained with appropriate role assignments to optimize performance. Ideally these should be performed *in situ*, i.e., embedded within the clinical environment. The frequency of simulation will vary according to the number of ECPR events managed by the team, however, when events are infrequent, we recommend at least monthly simulated ECPR training.

The review process should include the following components:

1. Hot debrief immediately after ECPR has been deployed to assess acute concerns or problems, and in particular, to acknowledge what went well.
2. ECLS daily rounds are important to assess the day to day management of the circuit and trajectory of the patient
3. ECLS monthly meeting are necessary to evaluate all aspects of the care provided and assess where improvements may be needed. These meetings should be structured with data measuring concerns for cannulation and complications with the circuit, anticaogulation, and patient-related complications including neurologic injury.

### Involving parents

A key role during any ECPR event is to faciliate information to the parents or care-givers of the patient. While the team has to focus on the phases of resuscitation and ECPR as described above, it can be very upsetting for parents to witness these events, or worse, not have information conveyed to them. Therefore, asigning an experienced individual to be with the parents and explain what is being done to their child is a priority. As to whether or not parents are present throughout depends on the team and the instiutional policies in this regard. In our experience we try to ensure parents are present during the phase 1 and 2, but once the surgical procedure starts and the equipment builds up in the confined space of our unit, we usually ask the parents wait outside of the area, and at the same time make sure there is ongoing line of communication with them during the procedure.

## Concluding comment

The utility of ECPR has been demonstrated in single center and registry retrospective studies, but as is often pointed out and concluded in various studies and reviews, there have been no randomized trials performed to definitively prove the benefit of ECPR over conventional CPR. The recommendations for ECPR have been couched in impartial terms such as “consider” or “it is reasonable”; there may be benefit, but no definitive recommendations.

It is our view from over 40 years of combined experience, that the existing data in a subset of children after congenital heart surgery and in children with reversible heart disease such as acute fulminant myocarditis, conclusively supports the notion of a system for ECPR as a requirement for any program undertaking pediatric cardiac surgery. This being the case, then the systems and people must be in place for effective resuscitation and timely implementation of ECPR, follow up of the patients and their families, no matter the size of the program.

## Author contributions

PL and A-MG shared equal contributions to the preparation of this manuscript, including review of relevant literature and citations, development and writing of content, and the opinions expressed within.

### Conflict of interest statement

The authors declare that the research was conducted in the absence of any commercial or financial relationships that could be construed as a potential conflict of interest.
